# Pannexins in the heart: cell-specific expression and contributions to disease

**DOI:** 10.1007/s00441-026-04046-9

**Published:** 2026-01-22

**Authors:** Mark C. Renton, Meghan W. Sedovy, Amanda Reynolds, Adam Hoch, Kailynn Roberts, Renée Sarmiento, Caroline Toler, Scott R. Johnstone

**Affiliations:** 1 The Fralin Biomedical Research Institute at Virginia Tech Carilion, Center for Vascular and Heart Research, 4 Riverside Circle, Roanoke, VA 24016 USA; 2 Virginia Tech Carilion School of Medicine, Roanoke, VA 24016 USA; 3 Translational Biology, Medicine, and Health Graduate Program, Virginia Tech, Blacksburg, VA 24061 USA; 4 Department of Surgery, Virginia Tech Carilion School of Medicine, Roanoke, VA 24016 USA; 5 Department of Biological Sciences, College of Science, Virginia Tech, Blacksburg, VA 24061 USA

**Keywords:** Pannexin, Ischemic heart disease, Cardiomyopathy, Purinergic signaling, Drug repurposing

## Abstract

Heart disease is the leading cause of death globally. Although modern interventions have dramatically reduced the morbidity and mortality of heart disease, the lack of knowledge of key underlying mechanisms has limited the development of effective therapeutics. Pannexins encompass a group of three transmembrane channel-forming proteins best known for their role in purinergic signaling through the release of ATP. Pannexins, particularly pannexin 1 (Panx1), are expressed in multiple cell types throughout the heart and play a role in blood vessel regulation, immune cell recruitment and activation, and the response to ischemic injury. In this review, we analyze publicly available sequencing data to investigate the expression of pannexin proteins in human and mouse hearts at both tissue and single-cell levels. We provide a detailed review of the literature surrounding cardiac pannexin function in the context of both ischemic and non-ischemic heart disease. We then discuss the clinical use of drugs now known to target pannexin channels as a primer for the therapeutic potential of pannexins in cardiac dysfunction. Finally, we discuss the largest gaps in the current literature to guide future research.

## Introduction

Heart disease is the leading cause of death globally, predominantly driven by atherosclerosis and ischemic heart disease (Jaiswal et al. 2025). Despite constant improvements in healthcare, the burden of cardiovascular mortality is expected to continue to rise into at least 2050 due to an aging population (Chong et al. 2025). The latest report from the American Heart Association (Martin et al. 2025) shows that in the USA alone, cardiovascular disease (including hypertension) was present in 48.6% of adults over 20 years of age and contributed to almost one million deaths in 2022. The combined direct and indirect annual costs of this burden of heart disease were estimated at $417.9 billion US Dollars between 2020 and 2021. As such, new therapeutics for the treatment of cardiovascular disease are required to reduce current trends in morbidity and mortality.

Pannexins (Panx) are a family of three proteins (Panx1, Panx2, and Panx3) that form single membrane channels between the cytosol and the extracellular space (Panchin et al. 2000). Panx1 is the most ubiquitously expressed isoform of the family and is best characterized as an ATP release channel (Bao et al. 2004), but it is also permeable to a wide range of ions and molecules such as glutamate, spermidine, and calcium (Ca^2+^) (Narahari et al. 2021; Yang et al. 2020).

In this review, we first analyze cardiac pannexin expression using publicly available protein and mRNA sequencing data, highlighting Panx1 as the most ubiquitously expressed isoform across the heart. We then provide a detailed review of cardiac pannexin function in the context of both ischemic and non-ischemic heart disease, including mechanisms such as immune cell recruitment, cardiac fibroblast isolation, and metabolic changes. Finally, we discuss the clinical use of drugs, highlighting probenecid and spironolactone, now known to target pannexin channels in the treatment of cardiovascular disease as a primer for the therapeutic potential of cardiac pannexins.

## Methods

### Human heart bulk mRNA sequencing reanalysis (GSE116250)

Raw counts were downloaded from the NCBI Gene Expression Omnibus (GEO) using accession number GSE116250 (Sweet et al. 2018) and reanalyzed using the R statistical analysis program (v4.4.3). Genes without at least 10 counts in one sample were filtered to remove low-expression genes from downstream analysis. Raw counts were normalized using DESeq2 (v1.46.0), and the mean normalized counts were plotted in GraphPad Prism (v10.4.1). Normalized counts were assessed for normal distribution using a Shapiro–Wilk test, and outliers were identified using the robust regression and outlier removal (ROUT) test (*Q* = 1%). One-way ANOVA followed by Tukey’s multiple comparisons was performed for normal data. The Kruskal–Wallis non-parametric tests were used for groups that did not display a normal distribution. Statistical significance was set as a *P* value of less than 0.05.

### Adult mouse cardiac non-myocyte single cell mRNA sequencing reanalysis (E-MTAB-6173)

The raw count matrix was downloaded from the EMBL-EBI’s Single Cell Expression Atlas using the accession number E-MTAB-6173 (Skelly et al. 2018). The matrix was read as a Single Cell Experiment using the “scran” package (v1.34), and cells were removed if they met any of the following criteria: (1) < 1500 or > 12,000 total reads; (2) < 600 or > 4500 expressed genes; (3) > 15% mitochondrial reads as a percentage of total reads; or (4) > 15% red blood cell reads (Hba-a1, Hba-a2, Hbb-bs, Hbb-bt, Hbb-bh1, Hbb-y, or Hba-x) as a percentage of total reads. These thresholds conform to accepted single cell sequencing quality control norms and correspond to the lower and upper tails of total read and detected gene distributions across all cells and aimed to exclude potential doublets, low-quality/dying cells, and empty droplets containing only background mRNA. Counts were log transformed and normalized before conversion to a Seurat (v5.3) object. Transformation and dimensionality reduction were then performed using the SCTransform pipeline and Uniform Manifold Approximation and Projections (UMAPs) were generated using 30 principal components. Normalized pannexin expression was then plotted across UMAPs. To annotate cell clusters, nearest neighbors were calculated with 30 dimensions and a K parameter of 20. Cell clusters were identified using shared nearest neighbors (snn) with a resolution of 0.2. Upregulated cell cluster marker genes were identified using the “findMarkers” function in “scran” with the binomial test type. The top 20 marker genes were extracted and unbiased cell cluster annotation was performed using the Annotation of Cell Types (ACT) online platform (http://xteam.xbio.top/ACT/), with Species = Mouse, Tissue = Heart (Quan et al. 2023). Unbiased results were then confirmed by literature search of marker genes and edited where appropriate.

### Developing mouse cardiac single cell mRNA sequencing reanalysis (GSE230531)

Raw data was downloaded from GEO using accession number GSE230531 (Ren et al. 2023). Raw data from each individual sample was read as a Single Cell Experiment using the “scran,” and barcodes were filtered using “DropletUtils” (v1.26.0). For each sample, cells were removed if they met any of the following criteria: (1) < 4000 or > 40,000 total reads; (2) < 400 or > 5500 expressed genes; (3) > 15% mitochondrial reads as a percentage of total reads; or (4) > 15% red blood cell reads (Hba-a1, Hba-a2, Hbb-bs, Hbb-bt, Hbb-bh1, Hbb-y, or Hba-x) as a percentage of total reads. All samples were converted to Seurat objects and then merged into a single Seurat object. The object was then split by embryonic day for transformation. Transformation, plotting of pannexin expression, and cell type annotation were completed as above for the previous dataset. For annotation using the ACT, Species = Mouse and Tissue = Embryonic heart settings were used.

## Pannexins

### Pannexin structure and activation

The structure, cell biology, and activation pathways for pannexins have been expertly reviewed previously (Good et al. 2015; Illanes-González et al. 2025; Isakson 2017; Laird and Penuela 2021; Luo et al. 2024; O'donnell and Penuela 2023). The pannexin isoforms share four α-helical transmembrane domains, two extracellular loops, a single intracellular loop, and carboxyl (C) and amino (N) termini located in the cytoplasm (Ambrosi et al. 2010; Baranova et al. 2004; Penuela et al. 2007). All three pannexin isoforms have now been modelled using cryogenic electron microscopy, providing insight into both channel-forming and gating mechanisms (He et al. 2023; Hussain et al. 2024; Li et al. 2025; Mou et al. 2020; Zhang et al. 2023a, 2021). These studies found that all three pannexin proteins arrange as heptamers to form membrane channels. Although pannexin heptamers typically comprise a single isoform, less stable heteromeric channels can form (Ambrosi et al. 2010; Bruzzone et al. 2005; Penuela et al. 2009; Sanchez-Pupo et al. 2018). Whether these heteromeric channels form in certain disease states in physiological systems, and the potential functional outcome of this, is currently not known.

Pannexins are regulated by post-translational modifications, including glycosylation, nitrosylation, caspase cleavage, and phosphorylation, that regulate membrane trafficking and channel opening (Penuela et al. 2014b). Pannexins contain a single N-glycosylation motif on either the first extracellular loop (Panx2) or the second extracellular loop (Panx1 and Panx3) often resulting in the generation of various pannexin glycoproteins (Penuela et al. 2007, 2009; Sanchez-Pupo et al. 2018). Glycosylation tightly regulates membrane trafficking and is thought to prevent the formation of gap junctions with opposing pannexin channels (Penuela et al. 2007, 2009; Sanchez-Pupo et al. 2018). Both Panx1 and Panx3 channel activity can be inhibited by S-nitrosylation on cysteine residues, which acts as an effective off-switch to prevent excessive ATP release following activation (Lohman et al. 2012b; Penuela et al. 2014a). This function is prevalent in the vasculature, where nitrosyl groups from nitric oxide are naturally abundant (Lohman et al. 2012b). The Panx1 C-terminal can be cleaved by caspases 3 and 7 during cell death, creating a constitutively open channel (Chekeni et al. 2010; Chiu et al. 2017). The subsequent unrestrained ATP release acts as a “find me” signal from dying cells to recruit phagocytes and other immune cells for clearance (Harcha et al. 2021; Lohman et al. 2015).

Panx1 is phosphorylated at tyrosine (Tyr) 198 and Tyr308 by Src family kinases (Billaud et al. 2015; Iglesias et al. 2008). Both Tyr198 and/or Tyr308 (mouse nomenclature) phosphorylation are required for channel opening and ATP release in the central nervous system and in the vasculature (Delalio et al. 2019; Lohman et al. 2015; Metz and Elvers 2022; Weilinger et al. 2016, 2012). However, data suggest equivalent sites in humans (Tyr199 and Tyr309) are not phosphorylated under the same conditions (Ruan et al. 2024). A third tyrosine site, Tyr150, has been identified as regulating glycosylation and cellular localization in culture, and was predicted to be phosphorylated by Src (Nouri-Nejad et al. 2021).

Serine (Ser) and threonine (Thr) sites also regulate Panx1. Panx1 Ser206 phosphorylation by protein kinase A/Protein kinase G (Medina et al. 2021; Poornima et al. 2015), Thr302/Ser328 by protein kinase A (López et al. 2020), and Ser394 by Ca^2+^/calmodulin-dependent kinase II (CaMKII) (Poornima et al. 2015) have been validated. It must be noted that the Ser394 site in mice and rats is an asparagine in humans (Hornbeck et al. 2015), limiting translation to human contexts.

Regulation by phosphorylation in Panx2 and Panx3 is less clear and understudied, with only Panx2 Ser514 (Ultanir et al. 2012) and Panx3 Ser68 (Ishikawa et al. 2019) identified in biochemical studies. Given phosphorylation is one of the most abundant post-translational modifications (Sacco et al. 2012), and little is still known regarding pannexin phosphorylation, we used PhosphoSitePlus (v6.8.1) to predict potential phosphorylation sites and their upstream kinases (Hornbeck et al. 2015). The most detected Panx1 phosphorylation site from high-throughput sequencing was Ser182 (human nomenclature), a site conserved across humans, mice, and rats. Using the surrounding sequence motif VTENLGQs*LWEVSEs (where * denotes the phosphorylated site), kinase prediction highlighted Polo-like kinase 4 as the strongest candidate for phosphorylation of this site. Panx2 has had few hits in high-throughput sequencing, but the most abundant was Ser514. This motif (KKHARHFs*LDVHPYI) is also highly conserved across species and with members of the AGC family of kinases, particularly Large tumor suppressor kinase 2, as predicted kinases. Panx2 Ser514 phosphorylation was identified by nuclear Dbf2-related kinase substrate pull-down in neurons (Ultanir et al. 2012). Panx3 also has had few hits, with Ser338 the standout. Again, highly conserved, this site (LFLRANIs*ELIsFSW) is predicted to be phosphorylated by members of the CaMK family, which is likely given the role Panx3 plays as an endoplasmic reticulum channel (Ishikawa et al. 2011). These predictions do not account for protein structure; therefore, physical site access for phosphorylation must be considered. Further molecular studies are required to validate these predicted sites and robustly establish the basis of pannexin regulation under physiological conditions in humans, and how this contributes to heart disease.

### Pannexin expression in the heart

**Fig. 1:**
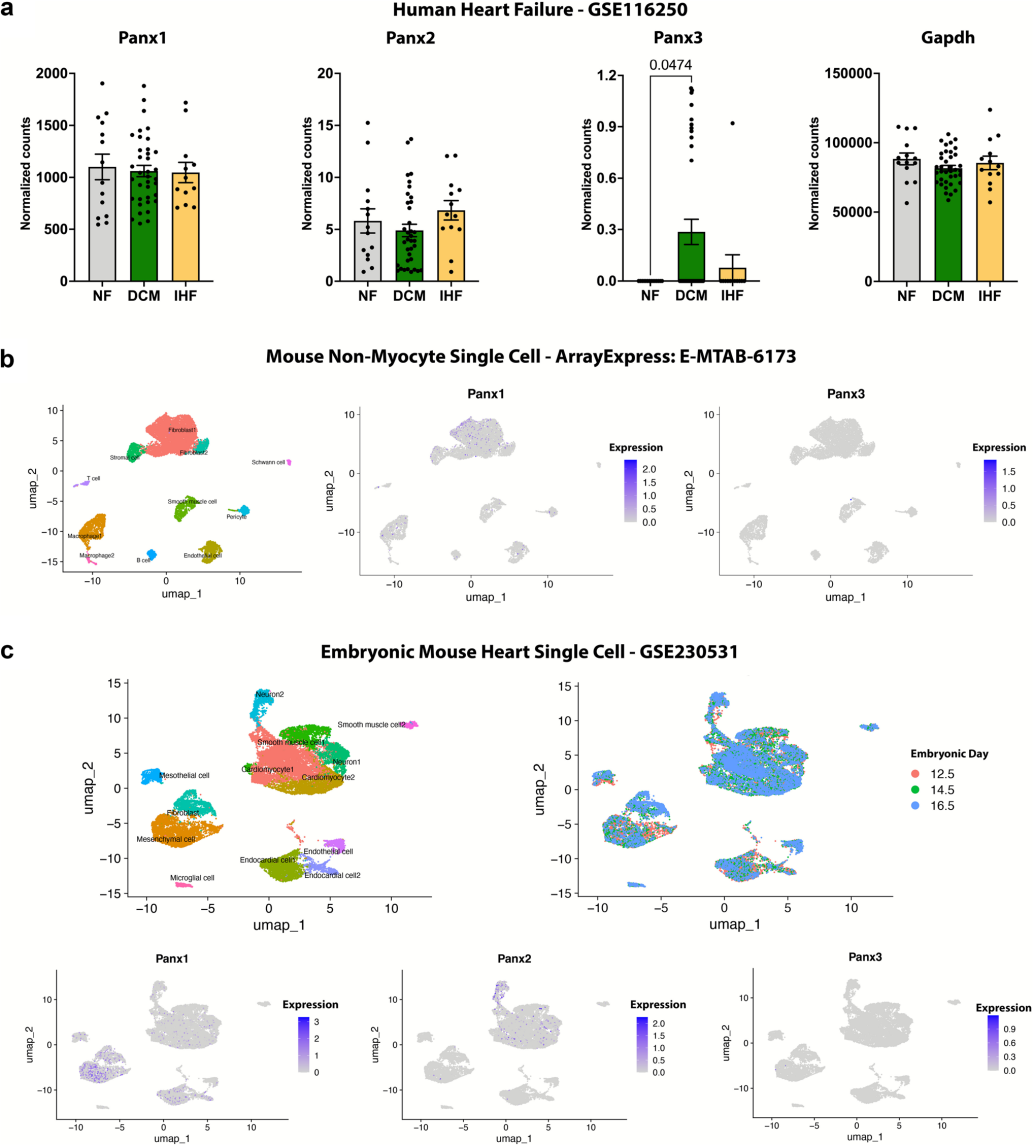
Panx 1 is the most ubiquitously expressed isoform in the human and mouse heart. Reanalysis of **a** bulk mRNA sequencing of human hearts from 64 patients of mixed sex with normal heart function (NF; *n* = 14), dilated cardiomyopathy (DCM; *n* = 37), ischemic cardiomyopathy (IHF; *n* = 13) (GSE116250) (Sweet et al. 2018). Normalized counts displayed as mean ± standard error of the mean. **b** Single cell mRNA sequencing of healthy C57BL/6J mouse (*n* = 4; 2 male and 2 female) cardiac non-myocytes (ArrayExpress E-MTAB-6173) (Skelly et al. 2018). Panx2 mRNA was not detected at this sequencing depth. **c** Single cell RNA sequencing of day 12.5, 14.5, and 16.5 embryonic hearts (*n* = 6; 2 per timepoint) from healthy C57BL/6J mice (GSE230531) (Ren et al. 2023)

Panx1 is ubiquitously expressed across many tissues, Panx2 mRNA is predominantly localized to the central nervous system but protein expression is more ubiquitous, and Panx3 is localized to osteoblasts, synovial fibroblasts, chondrocytes, skin, and vascular endothelial cells (Baranova et al. 2004; Le Vasseur et al. 2014; Moon et al. 2015; Wolpe et al. 2024). The heart is primarily made up of cardiomyocytes, fibroblasts, endothelial cells, smooth muscle cells, and immune cells (Kanemaru et al. 2023). To understand pannexin expression specifically in the heart, we analyzed publicly available transcriptomic datasets from both human and mouse hearts. We first analyzed a comprehensive human bulk RNA sequencing dataset of left ventricular tissue from 64 patients of mixed sex with normal heart function, ischemic cardiomyopathy, or dilated cardiomyopathy (Sweet et al. 2018) to gain a gross view of pannexin gene expression. Analysis shows that cardiac Panx1 expression is many magnitudes higher than Panx2 and Panx3 expression in human left ventricles (Fig. 1a). Interestingly, Panx1 and Panx2 gene expression did not differ between healthy and failing hearts. Panx3 had a statistically significant increase in expression in dilated cardiomyopathy compared to healthy hearts, but expression levels were still comparatively very low.

Bulk RNA sequencing does not reveal which individual cell types contribute to cardiac pannexin expression; as such, the abundance of cardiomyocytes in whole ventricle lysates may mask potential changes in expression in other cell types. With the advent of single-cell transcriptomics, uncovering cell-specific expression of genes has become much more feasible. We next analyzed the most comprehensive human heart single cell atlas currently available which sequenced 704,296 cells and nuclei from 25 adult healthy donors (14 female; aged 20–75 years old) (Kanemaru et al. 2023). This dataset revealed that Panx1 is expressed in essentially all cell types in the adult human heart, with the highest expression in activated fibroblasts and ventricular cardiomyocytes. Panx2 is expressed in some fibroblast clusters, but is otherwise not detected. Panx3 was not detected at this sequencing depth. This dataset (Kanemaru et al. 2023) can be visualized and downloaded at https://www.heartcellatlas.org/ where pannexin single cell expression across the human heart can be plotted.

Much of the current research on pannexins is in mouse models; therefore, we next investigated pannexin expression in a mouse heart single-cell RNA sequencing dataset. Given cardiomyocyte Panx1 expression is well established, we analyzed single-cell sequencing data of non-myocytes isolated from ventricular tissue from 4 (2 male and 2 female) healthy C57BL/6J mice (Skelly et al. 2018). Like the human data (Kanemaru et al. 2023), Panx1 mRNA expression was strongest in fibroblasts but was also expressed in all other cell types (Fig. 1b). No Panx2 transcripts were detected, and Panx3 expression was limited at this sequencing depth, matching the human single-cell RNA sequencing heart data (Kanemaru et al. 2023).

Finally, given adult pannexin mRNA transcript expression is difficult to detect using single cell sequencing, we looked at pannexin expression in developing mice. We reanalyzed a dataset that sequenced C57BL/6J mouse hearts at embryonic days 12.5, 14.5, and 16.5 (Ren et al. 2023). As in adult mice, after integrating samples, we found that Panx1 was relatively ubiquitously expressed and demonstrated the greatest expression in mesenchymal cells and fibroblasts (Fig. 1c). As expected, Panx2 demonstrated the highest expression in neurons but displayed some expression in cardiomyocytes. Panx3 mRNA expression was negligible at this sequencing depth. It must be noted that the lack of expression based on single cell data does not necessarily equate to the lack of expression in that particular cell type, as the sequencing depth of this analysis is still limited by current technology. Taken together, the data in human and mice clearly demonstrate that Panx1 is the most ubiquitously expressed isoform in the heart at the mRNA level.

Transcriptomics can give an indication of expression patterns, but mRNA transcription and protein translation are not directly correlated. Additionally, pannexins are relatively low-expressed membrane bound proteins, making false negatives a possibility in mRNA sequencing datasets (Isakson 2017). As such, it is important to understand the protein expression of pannexins across the heart. Panx1 protein expression has been demonstrated through western blotting, immunofluorescence, and flow cytometry in whole heart lysates and isolated cardiac cells from various animal models (Dolmatova et al. 2012; Kristiansen et al. 2018; Molica et al. 2017; Pavelec et al. 2024). Using western blotting in mice, Panx2 protein expression was found in every tissue tested in one study, including strong expression in the heart (Le Vasseur et al. 2014). Panx2 mRNA, measured by real-time qPCR, showed no significant correlation with Panx2 protein, furthering cautions against relying solely on mRNA data to quantify pannexin expression (Le Vasseur et al. 2014).

Western blot and immunofluorescence data in human hearts is currently limited, but immunohistochemistry using validated antibodies in the Human Protein Atlas (Uhlén et al. 2015) reveals medium cardiac Panx1 protein expression and no Panx2 expression, while there is no data on Panx3. However, the sections stained are cardiomyocyte heavy, leading to low resolution for other cell types. Human heart high-throughput proteomics may reveal further information. However, proteome coverage remains an issue in this field, with no pannexin peptides detected in two separate large-scale human heart transcriptomics screens (Doll et al. 2017; Linscheid et al. 2020). This is similar in mouse hearts with no detected pannexin peptides in several mouse heart proteomic datasets (De Freitas Germano et al. 2021; Edwards et al. 2023; Fang et al. 2025; Linscheid et al. 2021; Mohanty et al. 2025). Even if pannexin protein expression could be detected in proteomics data, this would be currently limited to whole heart or ventricle tissue, with current single-cell proteomics technology in its infancy.

## Pannexins in heart disease

The first known cardiac role for pannexins came from an electrophysiology study which documented Panx1 as a Ca^2+^-activated large conductance cation channel in isolated cardiomyocytes, suggesting a function in cardiac contraction (Kienitz et al. 2011). Since this seminal study, Panx1 has been implicated in the pathophysiology of both ischemic and non-ischemic heart disease in cell and animal models (Good et al. 2021; Pavelec et al. 2024; Rusiecka et al. 2023; Zhang et al. 2023b).

### Panx1 in myocardial infarction and ischemia/reperfusion injury

Myocardial infarction leads to a loss of oxygen and critical nutrients to the working cardiac muscle (ischemia), leading to cellular death. Despite restoring blood supply, reperfusion of the myocardium leads to further inflammation and damage. Ultimately, the irreparable muscle loss can result in heart failure and death. The mechanisms leading to both the acute damage during ischemia, and the subsequent complications of reperfusion are not fully understood, but purinergic signaling is thought to be heavily involved (Zhuang et al. 2023). Panx1, but not Panx2, gene and protein expression increases in response to coronary ischemia/reperfusion injury (I/R) in vivo in Sprague Dawley rat (Kristiansen et al. 2018) and canine (Dolmatova et al. 2012) hearts, hinting at a potential role in the cardiac injury response.

The involvement of Panx1 in coronary I/R was identified in studies on ischemic pre- and post-conditioning in the Langendorff-perfused male Sprague–Dawley rat heart (Vessey et al. 2010, 2011b, 2011a). These experiments showed that Panx1 formed channels alongside the purinergic receptor ion channel P2X_7_ during ischemic pre- and post-conditioning to secrete cardioprotectant factors such as sphingosine-1-phosphate and adenosine. Blocking Panx1 channels using CBX or Mefloquine completely blocked the cardioprotective effects of ischemic pre- and post-conditioning. Despite implicating Panx1 in the I/R injury process, these early studies were limited by ex vivo preparations and pharmacological agents that lack specificity to Panx1. This has since been addressed using genetic Panx1 knockout mouse models, where Panx1 has been shown to augment both the acute and chronic phases of I/R injury (Good et al. 2021; Rusiecka et al. 2025, 2023).

Panx1 contributes to myocardial injury and inflammation in the acute phase within 24 h of coronary reperfusion in male mice (Rusiecka et al. 2025, 2023). Global Panx1 knockout mice display a trend for reduced infarct size and significantly reduced serum cardiac Troponin-I levels, while functional cardiac measures were improved compared to wild-type control mice (Rusiecka et al. 2023). Given the global knockout, the contributing cell type(s) were not revealed. However, a constitutive neutrophil-specific (Ly6G-Cre) Panx1 deletion had no effect on I/R injury response, suggesting other cell types as key mediators (Rusiecka et al. 2023). A Panx1-targeting nanobody approach resulted in improved I/R injury response, indicating a potential pathway to clinical treatment outside of less-specific inhibitors (Rusiecka et al. 2025). In this study which produced a newly synthesized panel of Panx1-targeting nanobodies, male C57BL/6J mice treated with the nanobody Nb1 (20 mg/kg body weight) 5 min prior to reperfusion displayed significantly improved survival rates at 24 h post-I/R compared to control-treated mice, with no change in infarct size. In contrast to these studies (Rusiecka et al. 2025, 2023), in male C57BL/6J mice undergoing coronary I/R, a single dose (1.1 mg/kg) of probenecid at the onset of reperfusion had no effect on infarct size or the decrease in ejection fraction at 24 h post-I/R (Good et al. 2021). Using a tamoxifen-induced and endothelial cell-specific (Cdh5-Cre) Panx1 knockout mouse model also had no effect on infarct size or ejection fraction (Good et al. 2021), suggesting that endothelial Panx1 is not the sole contributing mechanism to the improved responses to acute I/R injury seen in the global Panx1 knockout (Rusiecka et al. 2023). However, differences in the genetic manipulation of Panx1, conditional versus constitutive, inhibit direct comparisons between the studies.

Panx1, specifically in endothelial cells, contributes to the chronic I/R response that leads to heart failure (Good et al. 2021). Further deterioration of cardiac function to that seen at 24 h post I/R was prevented in both probenecid-treated mice and endothelial cell-specific Panx1 knockout mice, with a significantly higher ejection fraction compared to control mice at 14-days post-I/R (Good et al. 2021). This protection coincided with a decreased recruitment of CD45^+^ Ly6C^HI^ proinflammatory macrophages in hearts 2-days post I/R. These results indicate that endothelial cell Panx1 is critical to the chronic deterioration of cardiac function post-ischemia/reperfusion injury, but not in the acute injury phase in male mice, likely due to the progressive recruitment of proinflammatory immune cells through released ATP.

It is important to note that I/R research in the heart has been performed solely on male mice and rats. Research into female mice is warranted, given that genetic and pharmacological Panx1 inhibition improved stroke injury outcome in female C57BL/6 mice only, with no effects seen in male mice (Freitas-Andrade et al. 2017). Female mice displayed significantly higher expression of total Panx1 in injured cerebral tissue when compared to male mice, potentially explaining this sexual dimorphism (Freitas-Andrade et al. 2017). As such, exploring Panx1 expression and I/R response in female mice should be a priority in this field.

Cardiac ischemia is typically the result of atherosclerotic plaque occlusion or rupture in the large coronary arteries (Libby et al. 2013). In male mice deficient of Apolipoprotein E, the most common murine model of atherosclerosis, endothelial and monocyte-specific (Tie2 Cre) Panx1 deletion increased the formation of atherosclerotic plaques in carotid arteries (Molica et al. 2017). Interestingly, this phenotype was lost in Apolipoprotein E deficient mice with global constitutive Panx1 deletion, likely due to metabolic alterations that were not present in the conditional Panx1 knockout model (Molica et al. 2017). This further highlights the functional differences that Panx1 plays in different cell types across the heart and body.

Taken together, these data suggest that Panx1 is upregulated in response to coronary ischemia and contributes to the pathophysiology of I/R injury in male mice, with female data currently lacking. Although the contributing cell types in acute I/R injury are unknown, endothelial Panx1 appears to be critical for the chronic injury response. In contrast, Panx1 is protective both in the initial development of atherosclerosis and during pre- and post-conditioning to coronary ischemia. We speculate that cardioprotective factors released by Panx1 in ischemic conditioning (Vessey et al. 2010, 2011b, 2011a) may prime surrounding cells for an ischemic event, potentially by promoting cell survival pathways. Whereas the inflammation and immune cell recruitment promoted by Panx1 ATP release following I/R contributes to injury and disease progression. Indeed, Panx1 is sensitive to inflammation, with a dramatic increase in Panx1 gene and protein expression following TNFα treatment in human endothelial cells leading to ATP and IL-1β release (Yang et al. 2020).

### Panx1 in non-ischemic heart disease and hypertrophy

Non-ischemic heart disease comprises conditions such as dilated cardiomyopathies, hypertrophic cardiomyopathies, metabolic cardiomyopathies, myocarditis, arrhythmias, and many others with varying etiologies (Wang et al. 2023). Typically, these conditions eventually result in heart failure or sudden cardiac death. Research exploring Panx1 function in non-ischemic heart disease is currently in its infancy, mainly focusing on β-adrenergic agonist-induced cardiac hypertrophy (Pavelec et al. 2024; Zhang et al. 2023b), providing a novel field of research to explore in future studies. Like ischemic heart disease, evidence for Panx1 signaling in non-ischemic heart disease is male-dominant, with a more concerted effort required for translation to female models.

Isoproterenol is a pan-β-adrenergic agonist that can induce cardiac hypertrophy and cardiomyocyte death at sufficient dosage (Chang et al. 2018). In isolated adult mouse cardiomyocytes, 10 μM isoproterenol for 30 min resulted in increased cellular death, ATP release, and dye uptake which were dependent on protein kinase A-mediated phosphorylation of Panx1 at Serine 206 (Zhang et al. 2023b). Furthermore, viral overexpression of a Panx1 construct with a single-point alanine mutation at Serine 206 in C57BL/6J mice blunted the acute (24 h) injury response to a 100 mg/kg dose of isoproterenol, as observed by a reduced immune response and plasma cardiac Troponin-I levels (Zhang et al. 2023b). During prolonged 14-day and 28-day isoproterenol regimes at 15 mg/kg/day, constitutive Panx1 knockout specifically in cardiomyocytes (Myhc6 Cre) protected mice from hypertrophy and cardiac dysfunction (Pavelec et al. 2024). Panx1 knockout mice also displayed reduced cardiac neutrophil burden at 14 days post-isoproterenol, while macrophage and dendritic cell accumulation was unaffected (Pavelec et al. 2024). Panx1 knockout cardiomyocytes also displayed increased glucose uptake and glycolysis, suggesting that a reduction in inflammation and a shift from oxidative phosphorylation to glycolysis may underpin the cardioprotective effects of Panx1 deletion in isoproterenol-induced hypertrophy.

Atrial fibrillation is the most common form of cardiac arrhythmia and can be induced by inflammation and vascular dysfunction (Kornej et al. 2020). Using mefloquine, spironolactone, or the specific Panx1 targeting peptide PxIL2P, which is designed against the Panx1 Tyrosine 198 motif (Koval et al. 2023), Panx1 blockade reduced the duration of adrenaline and caffeine-induced atrial fibrillation in mice treated with a single dose of vascular endothelial growth factor (Mezache et al. 2023). This suggests that Panx1, found in the coronary vasculature, may play a role in stress-induced arrhythmias.

Panx1 is also involved in cardiac dysfunction caused by metabolic disturbances, with cardiomyocyte Panx1 gene and protein expression significantly increasing in the hearts of male obese and type II diabetic *db/db* mice when compared to controls (Sun et al. 2023). In cultured primary mouse neonatal cardiomyocytes, high glucose media concentration (40 mM) resulted in an increase in ATP release that was blocked by treatment with Panx1 siRNA, highlighting a potential mechanism (Sun et al. 2023). However, whether a cardiomyocyte-specific Panx1 knockout could protect mice from high-fat diet or diabetes-induced cardiac dysfunction in vivo is unknown.

Infection, such as from SARS-CoV-2, can lead to myocarditis and associated cardiac dysfunction through both indirect mechanisms and direct infection of cardiomyocytes (Nappi and Avtaar Singh 2023). Purinergic signaling is a potential mechanism, as human induced pluripotent stem cell-derived cardiomyocytes incubated with the SARS-CoV-2 spike protein upregulate ATP release in a Panx1-dependent manner (Kato et al. 2022). The authors suggest that ATP released from Panx1 channels stimulates ACE2 production and viral entry into cardiomyocytes via a TRPC3/Nox2 regulated pathway (Kato et al. 2022). However, this work was performed in vitro in cultured cells, and whether Panx1 potentiates viral entry into the myocardium in vivo remains to be tested.

### Panx1 in cardiac fibrosis

Cardiac fibrosis, characterized by deposition and remodeling of the cardiac extracellular matrix, is a consequence of both ischemia/reperfusion injury and non-ischemic heart failure and leads to impaired contractility and relaxation (Frangogiannis 2021). Although the direct mechanisms initiating cardiac fibrosis are not completely understood, the activation of cardiac fibroblasts into myofibroblasts, which are characterized by high expression of smooth muscle actin, is critical. Activation of purinergic receptors by released ATP can activate myofibroblasts and stimulate profibrotic signaling pathways (Dolmatova et al. 2012; Lu et al. 2012). Given fibroblasts are share proximity with Panx1 channel-containing cardiomyocytes, infiltrating immune cells, and coronary vasculature cells, it is likely that ATP release from Panx1 channels contributes to fibroblast activation. Indeed, in an in vitro transwell coculture system, culture of isolated murine embryonic fibroblasts with hypoxic HL-1 murine atrial cardiomyocytes led to increased smooth muscle actin expression compared to fibroblasts cultured with normoxic cardiomyocytes, which was completely blocked by treatment with probenecid (Dolmatova et al. 2012).

Mechanical stress induces cardiomyocyte ATP release, with stretch inducing a rapid increase in extracellular ATP in neonatal rat cardiac myocytes, which was attenuated with CBX treatment or genetic Panx1 silencing with siRNAs (Nishida et al. 2008). Mechanically stimulated cardiac fibroblasts also release ATP as a self-activating purinergic signal to transition to a myofibroblast state (Lu et al. 2012). Cardiac fibroblasts isolated from adult male rats rapidly release ATP upon cell expansion from osmotic pressure, which can be blunted in a dose-dependent manner by CBX and probenecid (Lu et al. 2012). However, unlike in neonatal rat cardiomyocytes (Nishida et al. 2008), Panx1 siRNA treatment did not affect ATP release from adult rat cardiac fibroblasts, suggesting factors other than Panx1 could regulate this response (Nishida et al. 2008). Finally, in vivo data from mice administered isoproterenol for 28 days at 15 mg/kg/day led to increased levels of Collagen 1 immunofluorescent staining in wild-type control mice that was partially prevented in cardiomyocyte-specific Panx1 knockout mice.

This evidence suggests that reduced cardiac fibroblast activation by released ATP may underpin the potential protective effects of Panx1 inhibition on cardiac dysfunction. It must be noted that much of the evidence for Panx1 involvement in cardiac fibrosis is currently limited to in vitro cell culture, making generalizations to the adult human or rodent heart difficult. Additionally, connexin channels play a role in ATP release in disease conditions and could be the target of pharmacological treatments (Dolmatova et al. 2012; Lu et al. 2012). This warrants further research into the regulation of cardiac fibroblast activation and cardiac fibrosis by Panx1 in vivo.

## Pannexin targeting drugs in human cardiovascular disease

The search for effective treatments in essentially all forms of cardiovascular disease is at its peak given heart disease remains the leading cause of global mortality. Several pharmacologic agents that have shown preclinical and clinical efficacy in treating cardiac dysfunction have been shown to inhibit pannexin channels (Koval et al. 2023). Panx1 specifically appears to be susceptible to a wide range of compounds previously thought to be highly specific to their target protein (Dahl et al. 2013). Aside from Panx1 targeting peptides which have yet to reach clinical success, evidence suggests that Probenecid and Spironolactone are the most selective of the pharmacologic pannexin inhibitors (Koval et al. 2023), and their therapeutic role in cardiovascular disease is a topic of ongoing research.

## Probenecid

Probenecid is an FDA-approved medication originally used to treat gout by blocking reabsorption of uric acid in the renal tubules (Steele 1971). Panx1 inhibition by probenecid was first established in the amphibian *Xenopus laevis* oocytes, and its utility as a Panx1 inhibitor is amplified by the limited effect it has on connexin gap junctions (Silverman et al. 2008). Several studies have begun to repurpose probenecid in the treatment of cardiovascular disease given its anti-inflammatory and inotropic properties (Onódi et al. 2023).

One week of 1000 mg twice daily probenecid treatment significantly increased fractional shortening and myocardial contractility in patients with low baseline systolic function (Robbins et al. 2018). Interestingly, probenecid increased cardiac contractility without inducing the typical noxious pathways stimulated by sympathomimetic drugs. In addition, the ongoing “Re-Prosper-HF” trial is exploring the repurposing of probenecid in the treatment of heart failure with reduced ejection fraction (HFrEF) (Rubinstein et al. 2022). This trial aims to assess systolic function, functional status, and self-reported outcomes in probenecid-treated (1000 mg orally, twice per day for 180 days) patients when compared to a placebo group. Clinical data extends to the pediatric and young adult population, with one study investigating the role of probenecid in the treatment of univentricular circulation, a congenital heart defect (Rubinstein et al. 2020). Patients ≥ 40 kg were given 500 mg oral probenecid twice daily, and patients < 40 kg were given 250 mg/twice daily or a placebo for 4 weeks. Systolic and diastolic longitudinal strain, a sensitive estimate of myocardial function, improved with probenecid treatment. Peak oxygen consumption during exercise increased appreciably, though not statistically significantly.

These trials highlight the growing evidence supporting the utilization of probenecid in the treatment of cardiovascular disease, which could link to important cardiac functions for Panx1. However, more research is required to establish a clear benefit of probenecid treatment and the subsequent Panx1 inhibition in human cardiovascular disease. Also, much of the current evidence comes from retrospective analysis of clinical trials in gout patients, where confounding variables important in cardiovascular disease may not be considered. Additionally, as gout is a risk factor for cardiovascular disease (Cox et al. 2021), the reduction of gout through treatment could itself be a confounding variable in assessing the role of probenecid in this context.

## Spironolactone

Spironolactone, a mineralocorticoid receptor antagonist used for the treatment of heart failure, hypertension, edema, and primary hyperaldosteronism, is another pharmacological agent known to inhibit Panx1 (Good et al. 2018). Spironolactone is widely used for the treatment of heart failure and is FDA-approved for the treatment of HFrEF, resistant hypertension, primary hyperaldosteronism, hypokalemia, and edema secondary to cirrhosis and nephrotic syndrome. The landmark 1999 RALES clinical study established the role of spironolactone in the treatment of severe heart failure (Pitt et al. 1999). HFrEF patients receiving 25 mg of spironolactone daily improved symptoms and reduced hospitalization for cardiac issues and all-cause mortality. Reduction in the risk of death and hospitalization persisted through the follow-up period, which was two years on average (Pitt et al. 1999).

The American Cardiology College and American Heart Association (ACC/AHA) guidelines recommend mineralocorticoid receptor antagonists, like spironolactone, for patients with symptomatic HFrEF (Heidenreich et al. 2022), but the use case in heart failure with preserved ejection fraction (HFpEF) is less clear. In the large scale, multisite clinical TOPCAT trial, HFpEF patients treated with spironolactone had no significant improvements in cardiovascular outcomes (Pitt et al. 2014). However, HFpEF is a disease of highly variable patient-to-patient etiology with complex and relatively unknown pathophysiology, and evidence suggests that spironolactone is beneficial for certain subgroups of patients (Kosmas et al. 2018). As such, a role for Panx1 in human HFpEF should not be ruled out based solely on this evidence.

The therapeutic effects of spironolactone are attributed to its blockade of mineralocorticoid receptors. However, spironolactone reduces blood pressure in mice and inhibits vasoconstriction in hypertensive human peripheral arterioles in a smooth muscle cell Panx1-dependent, but mineralocorticoid receptor NR3C2-independent manner (Good et al. 2018). As such, spironolactone treatment would also likely regulate aspects of coronary artery and cardiac function via a Panx1-dependent pathway. However, potential synergistic actions of Panx1 inhibition and mineralocorticoid receptor antagonism on spironolactone’s mechanisms of action in heart failure have yet to be concretely defined.

## Other molecules

Colchicine, another gout treatment, can interfere with ATP signaling (Marques-Da-Silva et al. 2011) and it has been reported anecdotally that colchicine inhibits Panx1 channels (Dahl et al. 2013). However, further evidence is required to prove definitively that colchicine can block Panx1 channels. Clinical trials repurposing colchicine in heart disease have found reduced cardiovascular mortality in patients with chronic coronary disease (Nidorf et al. 2020) and after myocardial infarction (Tardif et al. 2019). Trovafloxacin is a quinolone antibiotic shown to inhibit Panx1 (Poon et al. 2014). However, it is unlikely Trovafloxacin will be recycled as a therapeutic option for the treatment of cardiovascular disease as it demonstrates significant vascular and hepatocellular toxicity in humans (Kaden et al. 2023). Carbenoxolone is an anti-inflammatory drug used to treat gastrointestinal ulcers (Pinder et al. 1976). First shown to inhibit pannexin channels in *Xenopus* oocytes, Carbenoxolone demonstrates a potent ability to block Panx1 (Bruzzone et al. 2003; Michalski and Kawate 2016). The ability to inhibit Panx1 channels with carbenoxolone is complicated by its capacity to also block Connexin gap junctions (Davidson et al. 1986). Given Connexin 43 gap junctions are critical for the propagation of cardiac electrical signals for contraction (Rodríguez-Sinovas et al. 2021), their use in cardiac contexts have been limited.

Overall, several inhibitors of pannexin have shown promising preclinical and clinical results in cardiovascular disease, highlighting pannexins as exciting prospects for the future of cardiovascular disease treatment.

## Conclusions and future directions

This review, including the secondary analysis of publicly available sequencing data, concludes that Panx1 is the most ubiquitously and highly expressed pannexin across the heart. Panx1 is critical in the response to cardiac disease conditions such as I/R injury and hypertrophy, predominantly through its involvement in purinergic signaling. This data, combined with the emergence of potent Panx1 inhibitors being repurposed for the treatment of cardiovascular disease, highlights Panx1 as an exciting potential candidate for the development of new treatments in cardiovascular disease. Despite the reviewed evidence, there remain many gaps in the field, including, but not limited to the following: (1) How Panx2, and potentially Panx3, fit into this puzzle, with more detailed research required on cardiac pannexin protein expression; (2) Pannexin’s role in the coronary vasculature; and (3) How pannexins contribute to cardiovascular disease with a metabolic basis.

Panx1 has been the focus of this review due to higher cardiac expression and the availability of published research. Human data suggest lower Panx3 expression in whole heart samples (Sweet et al. 2018), but Panx2 is consistently expressed in the heart. Despite this, little is known of its cardiac functions. Although no evidence exists of this so far, cardiac Panx2 or Panx3 could be upregulated or post-translationally modified in certain cell types and disease contexts and play a role in pathophysiology. As such, future research into pannexin function in cardiovascular disease should not entirely rule out Panx2 and Panx3.

The heart is highly vascularized, and the coronary vasculature is critical for function in both health and disease. Much is known regarding the expression and function of pannexins across the peripheral vasculature (Good et al. 2018; Isakson 2017; Molica et al. 2018; Wolpe et al. 2024). Panx1 is highly expressed in endothelial cells in all orders of blood vessels and is also expressed in smooth muscle cells in the small resistance arteries that are regulate blood pressure (Lohman et al. 2012a). The Panx1 inhibitory effects of spironolactone were originally discovered in the vascular system (Good et al. 2018). Additionally, Panx3 deletion specifically in endothelial cells induces hypertension in mice, highlighting a vascular role (Wolpe et al. 2024). However, all vascular beds are unique, and very little is known regarding the function of pannexins specifically in the coronary vasculature. Given the emerging theory that the coronary vasculature is critical in the pathogenesis of heart disease (Bockus and Kim 2022; Frąk et al. 2022), further research into the role of pannexins in this context is warranted.

Links between Panx1 and cardiac metabolic function (Pavelec et al. 2024; Rusiecka et al. 2023), reveal a promising field for future study. Obesity is a key driver of whole-body metabolic disturbances, and is an independent risk factor for heart failure (Caleyachetty et al. 2017; Kenchaiah et al. 2002), leading to the term obesity cardiomyopathy as a distinct cardiovascular disease of metabolic and inflammatory origins (Ren et al. 2021). There are currently no effective treatments for obesity cardiomyopathy; therefore, it will be important to explore whether pannexins are involved in the etiology of this disease.

## Data Availability

All code used in the reanalysis of sequencing data is available upon request.
